# Optimization of a microarray for fission yeast

**DOI:** 10.5808/GI.2019.17.3.e28

**Published:** 2019-09-18

**Authors:** Dong-Uk Kim, Minho Lee, Sangjo Han, Miyoung Nam, Sol Lee, Jaewoong Lee, Jihye Woo, Dongsup Kim, Kwang-Lae Hoe

**Affiliations:** 1 Aging Research Center, Korea Research Institute of Bioscience & Biotechnology (KRIBB), Daejeon 34141, Korea; 2 Catholic Precision Medicine Research Center, College of Medicine, The Catholic University of Korea, Seoul 06591, Korea; 3 Data Analytics CoE, SK Telecom, Seongnam 13595, Korea; 4 Department of New Drug Development, Chungnam National University, Daejeon 34134, Korea; 5 Department of Bio and Brain Engineering, Korea Advanced Institute of Science & Technology (KAIST), Daejeon 34141, Korea

**Keywords:** bar-code, fission yeast, gene-deletion, microarray, tag

## Abstract

Bar-code (tag) microarrays of yeast gene-deletion collections facilitate the systematic identification of genes required for growth in any condition of interest. Anti-sense strands of amplified bar-codes hybridize with ~10,000 (5,000 each for up- and down-tags) different kinds of sense-strand probes on an array. In this study, we optimized the hybridization processes of an array for fission yeast. Compared to the first version of the array (11 µm, 100K) consisting of three sectors with probe pairs (perfect match and mismatch), the second version (11 µm, 48K) could represent ~10,000 up-/down-tags in quadruplicate along with 1,508 negative controls in quadruplicate and a single set of 1,000 unique negative controls at random dispersed positions without mismatch pairs. For PCR, the optimal annealing temperature (maximizing yield and minimizing extra bands) was 58℃ for both tags. Intriguingly, up-tags required 3× higher amounts of blocking oligonucleotides than down-tags. A 1:1 mix ratio between up- and down-tags was satisfactory. A lower temperature (25℃) was optimal for cultivation instead of a normal temperature (30℃) because of extra temperature-sensitive mutants in a subset of the deletion library. Activation of frozen pooled cells for >1 day showed better resolution of intensity than no activation. A tag intensity analysis showed that tag(s) of 4,316 of the 4,526 strains tested were represented at least once; 3,706 strains were represented by both tags, 4,072 strains by up-tags only, and 3,950 strains by down-tags only. The results indicate that this microarray will be a powerful analytical platform for elucidating currently unknown gene functions.

## Introduction

Molecular bar-code arrays facilitate the parallel analysis of thousands of biological samples through a microarray [1]. In particular, the unique 20-bp DNA bar-codes or tags in each deletion strain enable the individual fitness of thousands of deletion mutants to be analyzed from a single pooled culture. In principle, the change in the number of cells of interest within the pooled library is visualized by the hybridization between fluorescence-labeled PCR amplicons of unique molecular bar-codes and their cognate probes on the array. This provides a powerful system for identifying the genes required for growth in any condition of interest [2].

These arrays are well known for their use with the tagged strains of yeast gene-deletion collections. Deletion collections have been constructed for budding yeast [3] and fission yeast [4,5]. Budding yeast is the pioneer model organism for gene-deletion collections, followed by fission yeast. As the two types of yeasts are distant within a phylogenetic tree [6], they play complementary roles in the systematic elucidation of gene function [7].

Among arrays for budding yeast [8,9], the first version of such an array (TAG3) was constructed with a 24-µm feature for each probe. In response to technological developments, the original TAG3 was improved to the TAG4 array. In particular, the feature size was reduced to 8 µm with a capacity of ~100,000 (100K). Furthermore, mismatch pairs and antisense-strand probes of each tag were removed, because they were proven to be uninformative. According to an analysis of data reproducibility, at most triple 8-µm features were needed to equal the performance of a single 24-µm feature. To test the ability of the TAG4 array to accurately measure differences in tag abundance, researchers conducted a signal ratio analysis and derived a correction function to adjust distorted intensity values due to the saturation effect.

Herein, we present detailed information on the optimization process of fission yeast arrays by incorporating useful pieces of information from earlier budding yeast arrays [8-10]. The optimization process of the array can reduce the inevitable defects caused by the innate hybridization bias. This study will provide a solid platform for fitness profiling using microarray technology.

## Methods

### Oligonucleotides, medium, and DNA samples

All the synthetic DNA oligonucleotides were obtained from Bioneer (Daejeon, Korea). Yeast cells were cultivated in YES medium (0.5% yeast extract, 3% glucose, and appropriate amino acid supplements) at 30℃ unless otherwise stated, following the manufacturer’s instructions [11]. Genomic DNA from the fission yeast gene deletion library was extracted using the Quick-DNA Fungal/Bacterial kit (catalog #D6005, Zymo Research Co., Irvine, CA, USA).

### Gene deletion library of fission yeast

The gene deletion library used in this study was constructed based on the principle of homologous recombination, as previously reported [4]. In brief, for each strain the open reading frame was replaced and tagged by homologous recombination with a deletion cassette consisting of the *KanMX* module (the selectable resistance gene *KanMX4* and a pair of unique 20-mer molecular bar-codes (up-tag and down-tag) on both sides flanking the *KanMX4* gene) and its flanking homologous regions to the chromosome (RHG).

### Design of PCR primer pairs and gene-specific tags

**Fig. 1.**
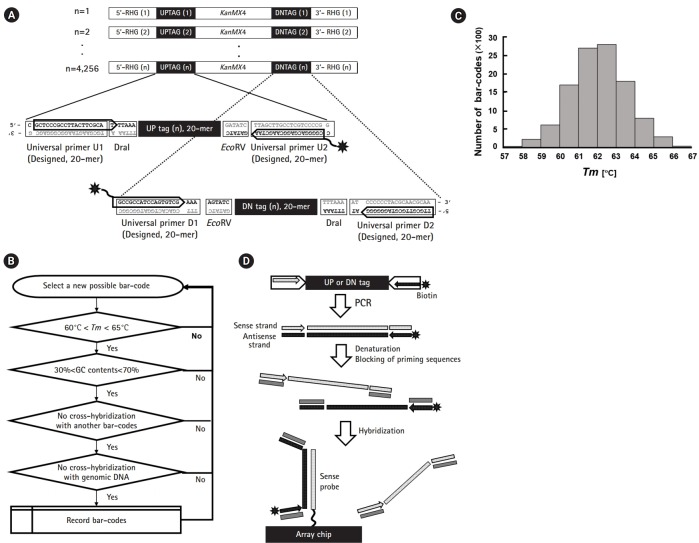
Schematic overview of the tag array in fission yeast. (A) Structure of deletion cassettes and detailed tag regions. The deletion cassette containing the *KanMX4* gene is flanked by a pair of unique bar-codes (UPTAG and DNTAG, corresponding to up-tags and down-tags, respectively) and regions of homology to the gene of interest (RHG). The deletion cassette replaces the open reading frame of interest by homologous recombination at the RHG regions. To facilitate whole genome analysis, each deletion cassette is assigned a molecular bar-code, a pair of unique 20-mer sequences, which are referred to as the “UP tag” and “DN tag” as shown in the insets with dotted lines. To amplify each tag for hybridization, PCR was performed using the following universal primer pairs: U1/U2 for the “UP tag” and D1//D2 for the “DN tag.” For labeling, biotin was attached to the U2 and D1 primers (shown as asterisks). (B) Scheme of bar-code design. The 20-mer bar-codes used in the study were selected using following criteria: (1) *Tm* of 60–65℃ with a deviation of ±2℃, (2) GC content between 30% and 70%, (3) less than 10-bp cross-hybridization with other bar-codes, and (4) less than 10-bp cross-hybridization with genomic DNA of fission yeast. The algorithm loop was repeated until to get 11,000 bar-codes. (C) *Tm* distribution of the bar-codes. The bar-codes obtained from the above algorithm showed a normal distribution in the range of 57–67℃ with a mean of 62℃. (D) Overall scheme of hybridization between tag amplicons and array probes. The pair of unique bar-codes consisting of an up-tag and a down-tag were amplified by PCR using a pair of flanking universal primers, one of which was labeled by biotin (shown with asterisks). The PCR product was hybridized with the custom-made array. Note that anti-sense strands (filled rectangles with white dots) of tags were hybridized to sense strands (dotted rectangles) of probes on the array. To reduce unwanted hybridization, all regions of the universal primers were shielded by blocking oligonucleotides (shown as gray rectangles).

Notable components of bar-code regions are represented in the schematic drawing shown in Fig. 1A. For amplification of up- and down-tags, pairs of each 20-mer primer, the universal primers U1/U2 and D1/D2, were theoretically designed (shown as two pairs of rectangles in the insets of Fig. 1A). Optimal PCR primer sets were empirically selected by the criteria of maximizing the yield and minimizing the extra bands (shown as two pairs of arrows, also refer to Fig. 3). The length of the four chosen primers was a 20/19-mer and a 17/18-mer for U1/U2 and D1/D2, respectively. For fluorescence detection of hybridization, biotin was linked at both ends of the anti-sense primers (shown as asterisks).

The sense strands of the bar-codes were designed for tiling on the array using the criterion of melting temperature (*Tm*, 60–65℃), GC content (30%–70%), and cross-hybridization with other bar-codes and genomic DNA regions (exact matches of no more than 10 bp, corresponding to a blast score lower than 20), as shown in Fig. 1B. Finally, ~11,000 bar-code sequences were selected through the above criteria with an average *Tm* of 62℃ (Fig. 1C).

### Hybridization: PCR and blocking oligonucleotides

Hybridization of the array was performed as previously described [4,12]. In brief, cells were collected during the pooled growth experiments, and their genomic DNA was prepared from frozen cell stocks. For each PCR sample, 10–20 OD_600_ (2–4×10^8^ cells/mL) were used. For amplification and labelling of gene-specific tags, PCR was performed with the indicated sets of universal PCR primers (as shown by the pairs of head-to-head arrows in Fig. 1A) using 0.2 µg of genomic DNA as a template. PCR amplification was performed through 30 cycles consisting of denaturation at 94℃, annealing at 55℃, and extension at 72℃ for 30 s for each step in a total volume of 100 µL (2.5 mM MgCl2, 0.2 mM dNTP, and 1 µM each primer mix). Hybridization was carried out using the Affymetrix Fluidics Station 450 (Pasadena, CA, USA).

As shown in Fig. 1D, only anti-sense strands of PCR products (shown as the filled rectangles with white dots) labeled with biotin (shown as the asterisks) were used for hybridization against sense-strand probes (shown as the dotted rectangles) tiled on the chip. In addition, for each tag, four priming sequences were shielded by blocking oligonucleotides (shown as rectangles in gray; refer to the previous report [4] for the sequence information), which prevented melted strands from re-associating.

### Design of the custom-made Affymetrix GeneChip

For the microarray experiments, two versions of Affymetrix GeneChips with 11 µm features were custom-made by Affymetrix, “Affy-KRIBB SP1 (Part No. 520429)” and “Affy-KRIBB SP2 (Part No. 520506),” by following the guidelines of the GeneChip CustomExpress Array Program.

**Fig. 2.**
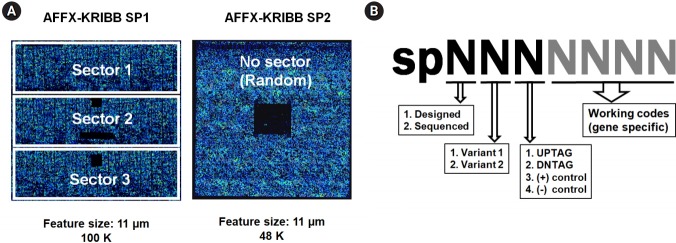
Design scheme of custom-made GeneChip and probes. For the microarray experiments, two versions of an Affymetrix GeneChip were made by a custom order. (A) Schematic overview of KRIBB-SP1 (100K) and KRIBB-SP2 (48K). The KRIBB-SP1 array was made to represent 43,721 probe pairs (perfect match and mismatch) in three separate sectors along with another control probes, corresponding to ~10,000 tags in triplicate or quadruplicate. The KRIBB-SP2 array was made to represent 41,169 probes without mismatch pairs at randomly dispersed positions along with other control probes, corresponding to ~10,000 tags in quadruplicate. For the reader’s convenience, array chips are represented in blue due to fluorescence, and the areas of negative control probes are represented by solid black squares within chips due to the absence of hybridization. (B) Strategy of probe design. Sequence information for each probe was represented in the sense strands by seven-digit numbers. The first digit represents whether its sequence was confirmed by Sanger sequencing, and the second digit specifies the type of deletion mutant. The third digit specifies the type of probe, including up-tag, down-tag, positive, or negative control. The remaining four digits represent working codes of deletion mutants, which replace complicated systematic ID numbers of each strain with simple numbers for the convenience of experiments.

Ideally, the first version (array format 100-3660) could represent 4,800 different probes by 11 probe pairs (perfect match and mismatch) with a maximum capacity of 100,000 (100K ≅ 4,800 × ×22). As the required features for the ~10,000 tag probe pairs in question (5,000 up-tags + 5,000 down-tags) exceeded 220K (10,000 × 22), the first version of the array was modified to represent 47,721 probe pairs in three separate sectors, consisting of 43,721 probe pairs (~10,000 tags in triplicate or quadruplicate, positive controls included) and a single set of 4,000 unique negative controls, resulting in 91K features (43,721 × 2 + 4,000) as shown in the left diagram in Fig. 2A. The second version (array format 400) was made to represent 48,201 features (48K), consisting of 41,169 probes for 10,292 tags in quadruplicate (~5,000 up-tags + ~5,000 down-tags) without mismatch pairs, 1,508 negative controls in quadruplicate, and a single set of 1,000 unique negative controls (~10,000 × 4 + 1,508 × 4 + 1,000) at randomly dispersed positions without separate sectors, as shown in the right diagram in Fig. 2A.

For tiling sense-strand probes on arrays, specific files containing the information about the total probes were generated in Excel spreadsheets, following the guidelines suggested by Affymetrix. Sequence information about each probe was represented in the sense strands by seven-digit numbers (Fig. 2B).

### Analysis of tag intensity and signal ratio

The probe intensity was obtained using the GeneChip Scanner 3000 and GeneChip Operating Software (Expression-Affymetrix MAS) with high-resolution upgrades. In brief, scanning of GeneChips generated a variety of data in multiple file formats, including .EXP, .DAT, .CEL, and .CDF. Among them, the.CEL files harboring probe intensity data were then analyzed using the R package ‘*affy*’ [13]. The signal ratio (Fig. 4) and the distribution of tag signals (Figs. 5 and 7 ) were plotted by kernel density plots, implemented in the R statistical software [14].

### Analysis of the relative growth rate

The relative growth rate (Fig. 6) was estimated from slopes of linear models with time measured in generations. The estimated data on the growth rates were added to the slope, which was set with a relative growth rate of 1.00 as the standard, as described previously [4].

## Results

### Optimization of bar-code PCR: annealing temperature

**Fig. 3.**
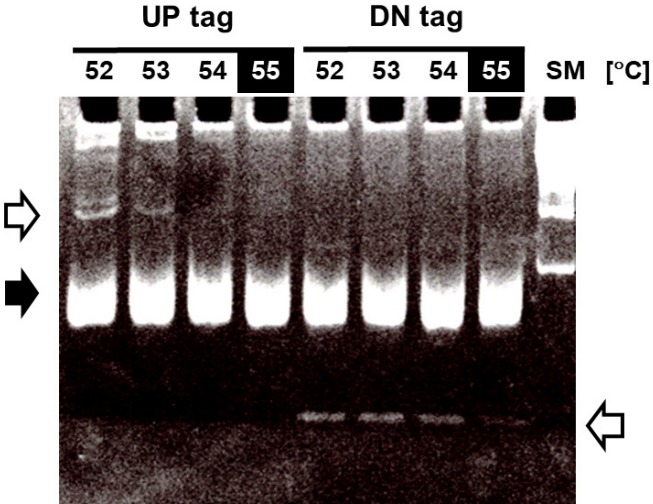
Optimization of the annealing temperature for PCR amplification of bar-code tags. To amplify the bar-code tags, the following sets of primers were used for PCR with 0.2 μg of genomic DNA as a template; UP tag, forward (5′U-2) 5′-GCTCCCGCCTTACTTCGCAT-3′, reverse (biotin-Kan5′U-2) 5′-biotin-CGGGGACGAGGCAAGCTAA-3′; DN tag, forward (DN3-F-biotin) 5′-biotin-GCCGCCATCCAGTGTCG-3′, reverse (DN3-R) 5′-TTGCGTTGCGTAGGGGGG-3′. To obtain the optimal PCR conditions, a series of PCR experiments were performed at the indicated annealing temperatures (52℃, 54℃, 56℃, and 58℃), followed by resolution on a 10% acrylamide gel with a size marker (SM, 100-bp DNA ladder from Bioneer). The arrows in white represent extra bands, and the arrow in black represents the position of amplified tag bands (~70 bp).

As the first step toward optimizing the GeneChip hybridization, the optimal annealing temperature of bar-code PCR was empirically determined using the criteria of maximizing yield and minimizing extra bands (Fig. 3). PCR amplifications for both the up-tags and down-tags were performed at the indicated annealing temperatures from 52℃ to 58℃, followed by resolution on 10% acrylamide gel in 0.5× TBE. The annealing temperature of 58℃ (shown in the filled squares on top) was best for both tags, which resulted in >30 ng yields at the position of 70 bp (shown by the filled arrow) with fewer extra bands (shown by the arrows at upper left and lower right). Notably, up-tag PCR showed a slightly higher yield than down-tag PCR, by ~10%.

### Optimization of hybridization: concentrations of blocking oligonucleotides

**Fig. 4.**
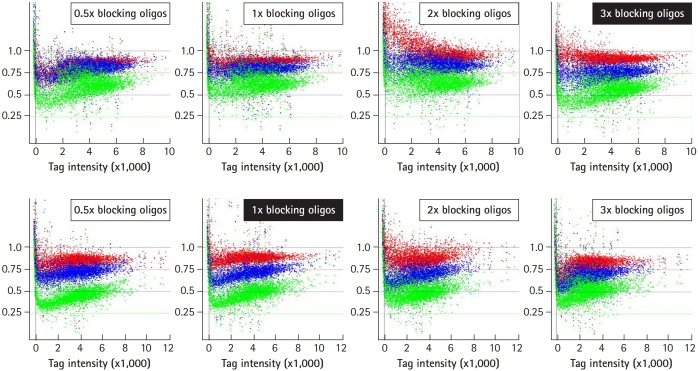
Optimization of signal ratios depending on the concentration of blocking oligonucleotides. The blocking oligonucleotides for each gene are required at eight primer sites for a pair of the four universal primers (U1/U2 and D1/D2). The concentration of blocking oligonucleotides (12.5 pm/µL each) was defined as 1×, corresponding to a 1:1 ratio of amplified tags. To determine the optimal concentrations of blocking oligonucleotides, a set of samples were hybridized with the indicated concentrations of blocking oligonucleotides from 0.5× to 3×, and subjected to analysis for the best resolution of the signal ratio. The dots in green, blue, and red represent each intensity from a variety of tag mixes, where up-tags comprise 25%, 50%, and 75% of the total mix and vice versa for down-tags. Note that 3× of up-tag and 1× of down-tag blocking oligonucleotides resulted in the best resolution of the signal ratio (shown as upper and lower filled rectangles).

To improve the ability of GeneChip to detect differences in tag abundance, eight different kinds of blocking oligonucleotides were used in hybridization. These blocking oligonucleotides help to keep single-stranded PCR products from nucleation or annealing with each other via universal primer sequences. To determine the optimal concentrations of blocking oligonucleotides, a set of samples were hybridized with the indicated concentrations of blocking oligonucleotides from 0.5× to 3× , and subjected to analysis of the signal ratio with a variety of the indicated tag mixes, including 25% (shown as green dots), 50% (blue dots), and 75% (red dots) for up-tags (upper panels) and *vice versa* for down-tags (lower panels). As shown in Fig. 4, the addition of 3× blocking oligonucleotides for up-tags clearly showed the best resolution of the signal ratio (shown by the filled rectangle in the upper panels). In contrast, addition of 1× blocking oligonucleotides for down-tags showed the best resolution of the signal ratio, as expected (shown by the filled rectangle in the lower panels). However, it is still difficult to explain why a higher amount of blocking oligonucleotides for up-tags than for down-tags was required for the best resolution of the signal ratio.

### Optimization of hybridization: mix ratio between up- and down-tags

**Fig. 5.**
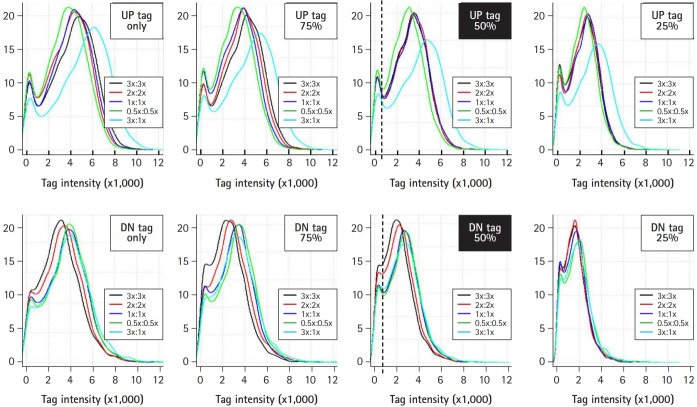
Optimization of the tag mix between up- and down-tags. For the best resolution of tag intensity, up- and down-tags were mixed at the indicated ratios with various concentrations of blocking oligonucleotides (shown as insets), and subjected to intensity analysis. A 1:1 mix between up- and down-tags resulted in the best resolution of tag intensity.

Next, up- and down-tags were mixed at the indicated ratios with various concentrations of blocking oligonucleotides and subjected to analysis for resolution of tag intensity. As represented by the upper and lower filled rectangles in Fig. 5, a 1:1 mix ratio between up- and down-tags showed the best resolution of tag intensity. Notably, a satellite peak was observed in the regions with a tag intensity of 200 from every set of samples (shown as vertical dotted lines), which corresponded to noisy signals (less than 4× the background signals) due to sequence similarity by chance. Thus, 85 deletion strains that presented noisy signals were eliminated from further array analysis.

### Optimization of culture conditions: temperature

**Fig. 6.**
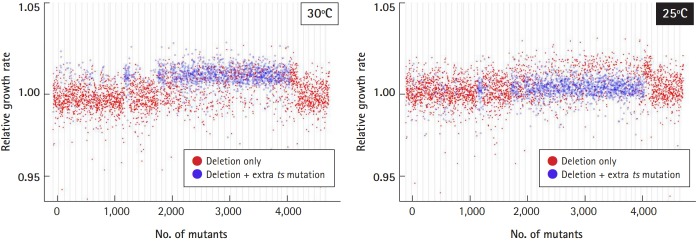
Optimization of culture temperature. To check the effects of temperature-sensitive mutants (*ts*-mutants) from a subset of the library on the growth profile, it was determined whether 25℃ or 30℃ would be optimal for cell cultivation. The relative growth rate of the deletion strains harboring extra *ts*-mutations (shown as blue dots) returned to normal compared to the normal deletion strains (shown as red dots) when cultured at 25℃.

As we reported previously [4], ~37% of the deletion collection harbored a recessive temperature-sensitive (*ts*) mutation unrelated to the gene deletion. Even though the entire set of essential heterozygous deletion strains (416) was remade, some viable heterozygous deletion strains still existed (1,400). As the mutations involved temperature sensitivity, we checked whether 25℃ or 30℃ would be optimal for cell cultivation (Fig. 6). The relative growth rate of the deletion strains harboring extra ts-mutations (shown as blue dots) deviated from that of the normal deletion strains harboring only targeted deletions (red dots), when cultured at 30℃ (left panel). However, the deviation of the growth profile returned to normal when cultured at 25℃ (right panel). Taken together, cell culture at 25℃ was found to be better than 30℃ if the deletion library is mixed with extra *ts*-mutants.

### Optimization of culture conditions: activation of the frozen pooled library

**Fig. 7.**
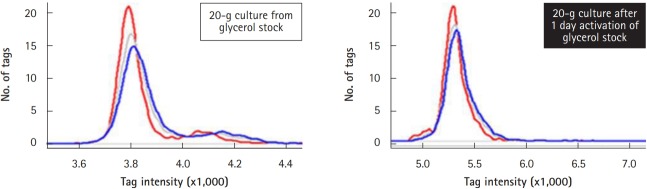
Optimization of intensity resolution by activation of frozen pooled cells. The effects of activation of frozen pooled cells on the resolution of tag intensity were identified in order to determine whether to activate the frozen pooled cells. The activation of frozen library cells for more than 1 day resulted in better resolution of tag intensity for up-tags (shown as the curved red line), down-tags (shown as the curved blue line), and the mean of both (shown as the curved gray line).

The heterozygous deletion mutant library was pooled, aliquoted into vials, and stored in a deep-freezer until use [12]. For systematic screening of target genes affected by drugs, a single vial was cultivated and treated with a drug. Cells were collected every five generations until 20 generations, and their genomic DNA was extracted for further microarray analysis. It was determined whether the frozen pooled cells should be activated for more than 1 day in order to obtain the best resolution of tag intensity. To do so, the resolution of tag intensity was compared between activated and non-activated samples (Fig. 7). When the number of up-tags (shown as curved red lines), down-tags (shown as curved blue lines), and total up/down-tags (shown as curved gray lines) were plotted against tag intensity using the same amounts of total intensity, the tag intensity of activated cells showed a better, broad distribution from 5.0 to 5.8 (right panel) in comparison to that of the non-activated cells (from 3.7 to 4.0; left panel).

### Summary of available bar-codes

Initially, 4,526 strains in total were pooled and entered into the optimization process. However, the bar-codes of 85 strains were eliminated due to the possibility of cross-hybridization, because they harbored a sequence similarity longer than 15 bp with each other by chance. This left 4,441 strains for further analysis. In addition, 125 strains showing noisy intensity for up-tags and/or down-tags were also eliminated, because they showed the intensity less than 4× background signals. Finally, 4,316 strains were proven to be useful for the microarray, as they were represented at least once among the up-tags and/or down-tags. In particular, 3,706 strains were represented by both tags, 4,072 strains by up-tags only, and 3,950 strains by down-tags only.

## Discussion

Molecular bar-code arrays enable the systematic identification of genes required for growth in any condition of interest [2]. These arrays are best known for their use with collections of yeast gene deletions, each of which is tagged with a 20-mer identifying DNA sequence known as a molecular bar-code. However, their extensive usage has been hampered by inevitable defects, such as intensity bias and bar-code mutations [15,16]. In a previous study [16], we reported that 16.6% of bar-codes contained mutations, as judged by a Sanger sequencing analysis of bar-codes. In the study, we optimized the hybridization processes of the array in order to reduce the inevitable defects originating from dozens of rounds of each hybridization step.

The universal primer pairs to amplify molecular tags were theoretically designed as 20-mer oligonucleotides. However, during the optimization process, it was elucidated that their actual lengths were a 20-mer/19-mer and a 17-mer/18-mer for U1/U2 and D1/D2, respectively. Furthermore, the annealing temperature was empirically obtained to obtain maximum yield with fewer noisy bands. In response to technological advances in microarrays, the second version of the GeneChip array was improved to show better performance without a change in feature size (11 µm) despite a twofold reduction in feature capacity from 100K to 48K. For example, the second version did not have separate sectors and mismatched probes, with fewer negative and positive controls. In contrast, the second version of the array in budding yeast [8] reduced the feature size from 24 µm to 8 µm, while retaining 100K features, but using a similar strategy to ours for probe allocation.

Compared with the optimization process of budding yeast arrays [8,10], a couple of optimization steps in fission yeast arrays are peculiar. Regarding blocking oligonucleotides, an intriguing observation was made that the concentration required for up-tags was three-fold higher than that required for down-tags. This unexpected phenomenon would make sense under the assumption that the PCR yield of up-tags would be higher than that of down-tags. When the DNA sequences of PCR primers were carefully checked, the universal primer D2 for down-tag PCR contained a “G” stretch, with six “G’s” straight in a row. This “G” stretch could result in a lower yield for down-tag PCR than for up-tag PCR. It was a mistake that we did not carefully check for “G” stretches inside the PCR primers. Next, a recessive *ts*-mutation unrelated to the gene deletion was contained in a subset of our deletion collection, which required an extra step to optimize the culture temperature.

Overall, 95% of the tags (4,316/4,526) in the deletion library were represented at least once among the up-tags and/or down-tags. At first glance, a 95% success rate appears good, but there might exist ~250 false-positive tags supposing a 5% detection error among ~5,000 deletions in fission yeast. To circumvent this problem that arises from both yeast collections, the problematic bar-code probes were corrected in the second version of the array as referred to earlier, as far as sequence data of the tags were available. However, the array technology still requires an upgrade to completely eliminate potential defects, which promises to make the fission yeast gene deletion library a reliable tool for understanding molecular gene function in a systematic way. In this regard, a platform based on next-generation sequencing (NGS) analysis would be innovative and would avoid potential errors, because each bar-code could be counted by direct sequencing irrespective of the inevitable defects caused by array hybridization. The results of this study serve as a basis for an innovative upgrade of the present microarray technology to a future NGS technology.
